# Reply to: Clinical utility of remote monitoring using multiparametric implantable defibrillators algorithm

**DOI:** 10.1002/clc.23961

**Published:** 2022-12-14

**Authors:** Federico Guerra, Giulia Stronati, Monica Campari, Sergio Valsecchi

**Affiliations:** ^1^ Cardiology and Arrhythmology Clinic Marche Polytechnic University, “Ospedali Riuniti” Ancona Italy; ^2^ Boston Scientific Italia Milan Italy

We would like to thank Dr Imamura for his interest in our paper and we have greatly appreciated his insights on the current perspective related to heart failure (HF) monitoring. In this reply, we would reply to his thoughts and clarify some aspects of our work.^1^ First, it is not entirely true that 44% “were not associated with heart failure‐related events” as this number more correctly represents the number of alerts not followed‐up by the physicians. These could have been false positives, as Dr Imamura correctly implies, nonactionable alerts (therapy changes or interventions already undertaken), or more simply true positives not properly followed by clinical response.^2^ In the first case, we are currently unable to assess the negative predictive value of the Heart Logic technology, although some conditions, such as SARS‐COV‐2 infections, were shown to trigger an alarm.^3^ In the last case, it has already been shown that the main issue nowadays is how physicians manage alerts, with many colleagues still waiting for the HF to become symptomatic, thus vastly undermining the potential for preventive action.^4^

In comparison to the CardioMEMS or other HF monitoring systems, the Heart Logic advantages rely mostly on its capability to condensate many clinical parameters (and not just filling pressures) into a single value, while giving the possibility to parcel out such value into its components, if deemed necessary. For example, if the patient's alert is mainly driven by an increase in the first and third sounds, an increased use of diuretics could be suggested. On the other hand, if the alert is mostly driven by an increased atrial fibrillation burden with high night heart rate, rate or rhythm control would probably be more successful. Another potential advantage is its integration with the standard of care ICD or CRT‐D, not needing a specific device to be implanted or a specific procedure to be reimbursed.

We agree with Dr Imamura that the next step in this journey would be how to integrate this technology with the new pharmacological therapies for HF, which have already been demonstrated to vastly change the neuro‐hormonal imbalance^5^ and provide many collateral benefits, such as a positive cardiac remodeling and arrhythmia reduction.^6^

In our work, we were not suggesting intervening by increasing diuretics exclusively. Indeed, of the 137 alert‐triggered actions, 56 consisted only of decongestive treatment augmentations while 81 were mixed interventions. Our findings agree with the recently published MANAGE‐HF study^7^ that showed a significant variability of response to alerts among centers. These results indicate a gap in the optimization of HF management that should be addressed to enhance guideline‐directed medical therapy with physiologic monitoring.

## CONFLICT OF INTEREST

M. Campari and S. Valsecchi are employees of Boston Scientific. The remaining authors declare no conflict of interest.
